# Proper care for the profoundly disabled depends on theology being recognized as queen of medical humanities

**DOI:** 10.1007/s40592-025-00247-0

**Published:** 2025-05-02

**Authors:** Charles Camosy

**Affiliations:** 1 Creighton University Medical Center, Omaha, USA; 2 St. Joseph’s Seminary and College, Yonkers, USA

**Keywords:** Theology, Bioethics, Disability, Moral status, Medical humanites

## Abstract

Despite the rise of the medical humanities in general in recent decades, theological bioethics—during a very similar period of time—was marginalized from many central bioethical discussions. This is problematic for a number of reasons. First, because theologians invented the discipline of bioethics and deserve a place at the table. Second, because the marginalization is often ideologically motivated. And third, because the vision of the human person that theologians bring to the table is essential for understanding the moral status of the proundly disabled.

The development of medical humanities over the past two or three decades has been a very significant one. As someone who at the time of this writing is teaching as a full professor at a medical school with an intensive ‘Gold Track’ for medical humanities required of all first- and second-year students (required class time is more than hours per week plus outside reading and writing—and a series of robust electives available to students who want to dive in more deeply), I’ve seen firsthand how the realization that “there is no view from nowhere” and that foundational, deeply-held values undergird the very nature of practice of medicine can shape future practitioners in foundational ways.

Unfortunately, I’ve also seen firsthand the dismissive attitude of some students—and, frankly, of far more current practitioners (and even medical school professors!)—who believe they have little or nothing to learn from the humanities. Firm in their convictions that it is only the white-coated class which has anything valuable to say about their discipline, and largely blind to the ways their foundational values (quite apart from anything they even learned in more traditional medical school courses) are guiding their practices, this significant number of folks close ranks and insulate themselves from the medical humanities discourse.

One could make an argument that an exception to this rule might be the relatively new prevalence of medical humanities discourses surrounding race, disability, and other matters (coded in some quarters as “DEI”: diversity, equity, and inclusion)—particularly when such discussions are related to broader discussions of social determinants of health. More attention has rightly been focused on how the contemporary practice of medicine has been compromised by economic and other social forces such that the human person has been divided up into specialty biological components and systems—the better to maximize income streams—with the result that care for the whole person in the fullness of who they are has been largely lost. Indeed, disciplines which focus on the whole person in this way (like primary care, family medicine, and geriatrics) are among the least lucrative and prestigious—regularly failing to match the necessary residents to meet demand from patients. This means that some of the most vulnerable populations, due to social forces pounding on them from multiple directions, rarely get the kind of care they need; instead, a system which has found algorithm-tested ways to maximize profits from our chronic disease epidemic is focused on ways to manage symptoms.

To the extent that medical students and practitioners can understand and really absorb these critiques, they are also aware that there is, again, no neutral view from nowhere when it comes to the values and paradigms at the center of their practice of medicine. They know they need to understand knowledge bases and academic methodologies different from the ones in which most of them trained: philosophy, theology, sociology, and more. In short: they need to understand medical humanities—and, as the need for an issue like this one makes clear, they have started to break through and make a real impact in certain circles.

Unfortunately, as both a bioethicist and Catholic moral theologian, I’ve seen the marginalization and retreat of theology from bioethics during the very decades that medical humanities have arisen to its new prominence (Camosy 2021).^1^ This is an astonishing fall, especially since Catholic theologians were inventors of the discipline of bioethics. Art Caplan—one of the most important figures in contemporary secular bioethics—acknowledges that, “truth be told”, today’s bioethics arose out of theology (Caplan 2015). Charles Curran also demonstrates that bioethics was already a well-developed subdiscipline of moral theology when secular bioethics arrived on the scene in the 1970s (Curran 2003).

Nevertheless, the marginalization and struggle of the current moment in bioethics is real—despite medical humanities becoming seemingly more prominent every year at the annual meeting of the bioethics-field-shaping American Society of Bioethics and Humanities. The last time I attended ASBH, I was dismayed (though, I hasten to add, not surprised) to see that not even a single paper had an explicitly theological argument. As I’ve pointed out elsewhere, those of us who do theological bioethics know that, in order to get a paper accepted for a formal ASBH session, one is forced to hide or translate one’s theological commitments into a secular idiom when submitting an abstract (Camosy 2021).^2^ The last time I was able to present (a paper on Christian theology and use of non-human animals in medical research) was the result of an invitation by the Christian theology interest group—the one place at ASBH (during the evening, apart from the formal sessions) where theologians can actually present and discuss theological sources and arguments which welcome their being explicit about the vision of the good which animates them.

But one might ask, why should secular academic organizations like ASBH allow formal papers from theologians? Aren’t theologians arguing from parochial perspectives that (at best) make such perspectives difficult for others who do not share their views to engage and (at worst) allow for backward and even dangerous and harmful views to be presented and taken seriously? Responses to such questions are quite easy to make—so easy, in fact, that one wonders why we should even engage them here. But the fact that they are so commonly asked, and the views behind them so widely held, suggest that they do require responses here. First (and once again), there is no view from a neutral nowhere when it comes to the values and paradigms engaged by medical humanities. Everyone—even those who are not explicitly religious—brings to bioethical discussions their particular and parochial visions of the good, ultimately supported by foundational first principles for which they do not have arguments. For the utilitarians, the foundational idea that we should maximize the greatest good for the greatest number requires no argument. For the human rights activists, the idea that we should treat all people fairly and equally similarly requires no case to support it. Requesting an argument for the idea from social justice activists that the most vulnerable must have special consideration also seems absurd. That’s because those who hold such positions do not hold them on the basis of arguments. They hold them because their truth grabs or claims them on the basis of intuition, faith, or some other kind of authority.

But a moment’s reflection reveals that these principles are not at all obviously true to everyone. On the contrary, many secular thinkers utterly reject one or more of these secular first principles. Indeed, though some thinkers have tried to make all three of these principles cohere, from many serious perspectives it is impossible to reconcile the idea that we can ultimately maximize good consequences, defend the fundamental equality of all, and have a preferential option for the most vulnerable (Appiah 2006). For those who think it is impossible, then thinkers who simply accept one of these principles as true—and use it as a foundation for an argument, say, in a paper at ASBH—are arguing from their parochial perspectives that (at best) make them difficult for those who do not share their views to engage and (at worst) allow for backward and even dangerous and harmful views to be presented.

But why, then, are secular utilitarians, secular human rights thinkers, and secular social justice activists nevertheless welcomed at conferences like ASBH and into the medical humanities discourse more broadly, while theologians (especially, but not only, in US contexts) are largely not? Part of the explanation surely lies in a kind of ignorance, perhaps born of soft bigotry: gatekeepers who hold power in these contexts may simply not have thought much or carefully about these matters and have an unthinking, reflexive, and socially-conditioned reaction to theology which leads them toward a marginalization (Callahan 2012). This, while regrettable, is at least morally excusable. What cannot be countenanced—and must be condemned—is similar behavior from those who know better.

Some of those who know better were the philosophers who came to prominence in bioethics right alongside theologians. Dan Callahan, writing in In Search of the Good: A Life in Bioethics, explains how some of these philosophers moved intentionally and quickly to “overshadow” and, eventually, simply to push aside the theologians (Callahan 2012). They did so in ways that were sometimes more subtle (using different language and concepts, sharply contrasting styles of argumentation, etc.), but at times they were more direct, with open hostility to religious ideas. The hostility between moral theologians and philosophers was so strong, Callahan notes, that when assembling bioethics research teams he spent much of his time managing the philosophers’ anger at having to engage with theologians (Callahan 2012).

But, as I note in my book Losing Our Dignity: How Secularized Medicine is Undermining Fundamental Human Equality, Callahan was fighting a losing battle (Camosy 2021). This secularizing, philosophical approach came to dominate what was read and used in the field. Tom Beauchamp and James Childress’s Principles of Biomedical Ethics, first published in 1979, and an eighth edition published in 2019, exemplifies this decades of dominance (Beauchamp and Childress 2019). Theologians close to both Protestant and Catholic bioethics (like Joseph Fletcher, Paul Ramsey, Charles Curran, and Richard McCormick) sometimes could not be distinguished from their secular counterparts because their perspectives included little or no explicit theological content (Jonsen 2006; Tham 2007). To remain at or near the top of the new discipline, or even merely to be welcome in it, they were forced to strip most of their theology out of their bioethical research and publication (Jonsen 2006).

This secularizing and even irreligious trend was also quite prominent throughout the broader academy. This shift in bioethics took place during a time when theology departments struggled to give an account of their place in the university and broader academic institutions and societies—and with the result that such departments began to morph into “religious studies” with professors who studied “social ethics.” Most explicitly theological ethicists have decided not to write on bioethics, and good universities are struggling even to find marginally viable candidates for excellent jobs in theological bioethics. Some of this shift could be attributed to careerism and/or cowardice, but some of the shift is totally understandable when some—especially someone with a family and mortgage—comes across philosopher Timothy Murphy’s “In Defense of Irreligious Bioethics,” a target article featured in one of the most prominent journals in the field: the American Journal of Bioethics (AJOB) (Murphy 2012). Murphy’s article, which has become widely influential—so much so that the Journal of Medicine and Philosophy planned an entire issue responding to it—argues that theologians and religious traditions involved in bioethics should face a “hermeneutic of suspicion” (Murphy 2012; Camosy 2021).^3^ Murphy’s directness was admirable, especially given that most of this marginalization had taken place previously only in ways that were largely hidden. He argued publicly that religious traditions should be singled out and repudiated in bioethics and even that hostility “may even be morally required” of secular bioethicists. Significantly, AJOB recently published a target article which argues for “re-establishing the relationship between theological and secular bioethics” (Stahl and Emanuel 2020). The working assumption here is that Murphy’s irreligious aspirations for bioethics have been successful and must be addressed.

Thinkers like Murphy, and those whose stories Charles Curran related to us, have clearly used power to set their ideological agenda and exclude theologians and theological ideas who do not fit with that agenda. We know this because sometimes an exception is made for certain theological perspectives when they fit with the agenda. As I argued in Losing Our Dignity, a secular activist for a migrant’s right to health care, for instance, generally won’t exclude Pope Francis’s ideas in favor of the full cultural inclusion of migrants merely because of the Pope’s clear theological commitments (Camosy 2021; Francis 2018). On the contrary, they are welcome allies. But a secular abortion activist, by contrast, will very much exclude ideas like those of Pope St. John Paul II’s concerning the full inclusion of prenatal human beings—precisely because of the pope’s clear theological commitments (John Paul II 1995).

But it is the concept of full human dignity and equality—on the basis of nothing other than being a member of the species Homo sapiens made in the image and likeness of God—that has been put at the most risk because of the marginalization of theological bioethics. The replacement of fundamental human equality with a “trait X” ethic (where X is something like autonomy, rationality, self-awareness, and the like) puts disabled human beings at serious and particular risk. Indeed, it is not too strong to say that the full moral and legal status of disabled human beings—and their proper care—may require theology to recover its prominence in the fields of bioethics and of medical humanities.

Many readers will be aware of the implications of fundamental human equality for debates over abortion. Though the clear humanity of fetal life is no longer under serious dispute, and the full moral status of a prenatal human being is nothing close to dispositive for the abortion argument (indeed, the most famous and influential pro-choice argument ever made presumes the full moral status of the baby) (Thomson 1971), many who want to defend abortion rights—which is the closest thing to a sacred cow in secular bioethics—simply reject fundamental human equality because it may put abortion rights at risk.

But rejection of fundamental human equality has other very significant implications beyond abortion, particularly for the profoundly disabled. The American Academy of Neurology recently claims that they have “consensus” on the claim that we can take organs from a dead patient, even if said patient still has a functioning hypothalamus and other biologically human coordinated responses (Lewis et al. 2023). Joe Fins has demonstrated that patients who are deemed to be in a so-called vegetative state are sometimes conscious and can be helped significantly by various therapies (Fins 2015). But his calls for a new civil rights movement for this population of human beings have been met with only prognostic pessimism and therapeutic nihilism.

But perhaps the most significant import for the profoundly disabled, given a rejection of fundamental human equality, is the grim future faced by those with dementia. Peter Singer rightly pointed out that, once we reject fundamental human equality, the rejection of the moral and legal status of those with dementia is not far behind (Spector 1999). These human beings lack in very significant ways the “Trait X’s” so prized by our post-human equality bioethical culture: rationality, self-awareness, autonomy, and more.

While we aren’t aiming at their deaths as human non-persons quite yet—as we are with prenatal human beings and those in a so-called vegetative state—we are getting closer to such a practice. Indeed, the way we treat many such human beings now is not much better than killing them. We largely remove these individuals from their homes, isolate them in poorly staffed institutions, keep them ‘engaged’ primarily via the television and increasingly by robot ‘caregivers’ and ‘friends’, and—in the face of care resource shortages—give them contraindicated antipsychotic drugs which double their chance of death and serve essentially as a chemical straitjacket (Schneider 2021).

And if anyone wonders about our future trajectory, think about the following example from the Netherlands. Taught by her culture that her life would have no value once her disease progressed, a Dutch woman with early-stage dementia requested physician-assisted killing (Kolata 2017).^4^ But she was not set to be killed, however, until the disease progressed further. Sickeningly, she woke up after taking the deadly drug in her coffee and, apparently resisting the killing process now underway, had to be physically restrained by the doctor and her family for the killing to be completed. The physician who used physical force against her patient in order to kill her was cleared of any wrongdoing in the matter (Kolata 2017).

If current trends stay the same, the number of people with dementia will double in the next twenty years and will triple in the next 30 (WHO 2017). Most Western countries already lack the will and resources to care for this population properly given the current numbers: what will happen over the next three decades as the numbers skyrocket? What will happen if theology continues to retreat and be marginalized from bioethics’ necessary conversations spurred by medical humanities—and, with it, the concept of fundamental human equality that would include dementia patients who have lost their rationality, self-awareness, autonomy, and/or similar “Trait X’s”? I argue it is highly likely that continued (1) resource scarcity and (2) ideological shifts away from fundamental human equality will mutually reinforce each other in a regressive devolution that will lead to the mass killing of dementia patients as human non-persons who are little more than burdens on those deemed to be full moral and legal persons. Indeed, when we aim at their deaths, our doing so will be understood similarly to the way we currently understand aiming at the deaths of prenatal human beings and those in a so-called vegetative state.

Though a handful of secular thinkers argue for something similar to a theological account of fundamental human equality, it is difficult to imagine stemming and reversing the forces pushing in this direction without recovering the prominence of theology and theologians in discussions of bioethics and humanities.

In the Middle Ages theology was known as the “Queen of the Sciences” (Talar 2020). This was due to several reasons—including the fact that ‘science’ in the era had a broader meaning, referring to any systematic study of knowledge. But primarily it was because theology was welcomed as the field concerned with ultimate questions and therefore the highest form of knowledge. Theology, as the field still primarily and explicitly concerned with first principles, foundational ideas, and sources of ultimate concern, ought to be given a similar space—but in this context as Queen of Medical Humanities.

This, of course, could not happen overnight. But, as mentioned above, a foundational shift in bioethics which recognizes the significant place of first principles, foundational ideas, and sources of ultimate concern would at least bring theology back into the fold as a respectable conversation partner at ASBH and in other significant bioethical contexts (McKenny 2023).^5^ This would not only right the wrongs of its marginalization during the past few decades, but also create the conditions for the possibility of theology taking its rightful place as Queen of Medical Humanities (Camosy 2021).^6^ Given our secular shift away from fundamental human equality, such a shift is likely to be the only reasonable hope for the proper care of profoundly disabled human beings who are well on their way to being deemed non-persons.

## Data Availability

No datasets were generated or analysed during the current study.

## Footnotes

1 When I say “theology” here, and most often throughout the text, I have in mind Christian theology. Not only because Christian theology was the Queen of Sciences in the Middle Ages and Christian theologians were the ones who invented contemporary Western bioethics (and were then marginalized), but also because it is the tradition I work in and know best. However, at least in principle, other theologians from other faiths could also play significant roles in the vision I’m outlining in this piece—not least because Judaism and Islam have similar understandings of human dignity and human equality, especially in relation to the disabled.

2 A colleague of mine in moral theology, new to bioethics, thought I must be wrong about this claim and decided to informally test it. He had submitted an abstract the previous year in which he translated out the theological content and ASBH accepted it. In testing the principle, he submitted one with explicit theological context and found his proposal rejected. It was only the third in his 20-plus year career that this happened to him.

3 In fact, though at the time this book goes to press it has not yet been published, the Journal of Medicine and Philosophy is poised to devote an entire issue responding to Murphy’s arguments.

4 This Dutch euthanasia case highlights how cultural attitudes toward disability and dependency can shape end-of-life decisions, raising profound ethical questions about autonomy and coercion.

5 There is some hope that we may be moving in this direction, as evidenced by recent discussions in bioethics welcoming religious perspectives.

6 Some may wonder how this might work, practically speaking, in the context of a new bioethics paradigm I’m proposing. This would need to be worked out over decades, but I could see two senses in which this came to be. First, theology could take its rightful place as Queen of Medical Humanities simply by the discipline moving toward a particular and foundational focus on first principles, foundational ideas, and sources of ultimate concern. This would be a significant shift away from the undue focus in recent years on short, multi-authored papers (often from clinicians with no training in these matters) which either skip over these central ideas or touch on them so briefly that they could hardly be said to engage them. A second way theology could take its rightful place as Queen of Medical Humanities could mirror the way that other approaches (principalism, utilitarianism, etc.) have become dominant in certain bioethical contexts. Perhaps with a focus on disability at the center, theological approaches could eventually become the default or primary way we imagine doing bioethics. As this article has shown, this was certainly the case when bioethics was invented as a discipline in the Western world, so with that example we have proof of concept.
